# Dissociating the sequential dependency of subjective temporal order from subjective simultaneity

**DOI:** 10.1371/journal.pone.0223184

**Published:** 2019-10-09

**Authors:** Renan Schiavolin Recio, André Mascioli Cravo, Raphael Yokoingawa de Camargo, Virginie van Wassenhove

**Affiliations:** 1 Centro de Matemática, Computação e Cognição (CMCC), Universidade Federal do ABC (UFABC), São Bernardo do Campo, SP, Brazil; 2 Cognitive Neuroimaging Unit CEA DRF/Joliot, INSERM, Université Paris-Sud, Université Paris-Saclay, NeuroSpin Center, Gif-sur-Yvette, Paris, France; Birkbeck University of London, UNITED KINGDOM

## Abstract

The physical simultaneity between two events can differ from our point of subjective simultaneity (PSS). Studies using simultaneity judgments (SJ) and temporal order judgments (TOJ) tasks have shown that whether two events are reported as simultaneous is highly context-dependent. It has been recently suggested that the interval between the two events in the previous trial can modulate judgments both in SJ and TOJ tasks, an effect named rapid recalibration. In this work, we investigated rapid recalibration in SJ and TOJ tasks and tested whether centering the range of presented intervals on perceived simultaneity modulated this effect. We found a rapid recalibration effect in TOJ, but not in SJ. Moreover, we found that centering the intervals on objective or subjective simultaneity did not change the pattern of results. Interestingly, we also found no correlations between an individual’s PSS in TOJ and in SJ tasks, which corroborates other studies in suggesting that these two psychophysical measures may capture different processes.

## Introduction

By living in a stream of continuous sensory stimulation, our brain has to infer whether incoming signals in different sensory modalities specify a common or a different cause in the external world [1–5]. A major principle that informs such decision is the spatial and temporal coincidence of sensory events: the closer in space and time multisensory events are detected to be, the likelier they will be considered to specify a common cause [6, 7]. In this context, understanding the rules by which simultaneity (favoring the *integration*) and temporal order (favoring the *segregration*) of multisensory events operate becomes essential. For instance, witnessing a glass breaking on the floor is naturally experienced by an observer as a single event, given the smashing and the noise co-occur in time and space; however, a lightning bolt and thunder are beyond our horizon of temporal simultaneity [8]. Indeed, the larger the viewing distance, the more delayed the sound needs to be for audiovisual (AV) events to be perceived as simultaneous: an increase of 3 ms of delay per meter was found with AV distance for up to 10 meters of distance suggesting the existence of compensatory mechanisms that may maintain simultaneity [9–11].

One difficulty in addressing such issues is that multisensory simultaneity has been investigated using two main protocols. First, there are studies investigating the perception of AV simultaneity using simultaneity judgments (SJ) [12–16], in which participants are presented with AV events separated by varying temporal delays (or stimulus onset asynchronies, SOAs) and have to estimate whether the sound and the visual stimulus were simultaneous. From the simultaneity curve, typically an asymmetric Gaussian sometimes presenting a plateau, one can derive the points of subjective simultaneity (PSS) corresponding to the SOAs at which participants consider the AV events to be simultaneous. Other studies have used temporal order judgment (TOJ) tasks [17–19], in which participants have to report which of the auditory or visual event was presented first. A psychometric curve allows inferring a “point of subjective simultaneity” (PSS) corresponding to an SOA at which participants are most uncertain about the order of events. Although both measures are taken as indices of simultaneity perception and referred to as “PSS”, it has long been noticed that they are likely the outcomes of different underlying mechanisms [20, 21].

Second, a series of studies have suggested that individuals’ PSS are context-dependent. In particular, the temporal recalibration phenomenon observed both in SJ [22, 23] and in TOJ tasks [23–25] showed that repeated exposure to specific AV delays can change a participant’s PSS. Additionally, a single trial was shown to be sufficient to generate AV temporal recalibration in an SJ task [26–29]. A recent study further suggested that rapid recalibration could be found in TOJ task as well [30]. In his study, Roseboom [30] suggested that rapid recalibration results in opposite effects for TOJ and SJ: in the SJ tasks, an asynchronous N-1 trial will influence the judgment of trial N towards being synchronous, corresponding to a negative after-effect; in the TOJ task, the reported temporal order in trial N tends to be the same as the perceived order in trial N-1, corresponding to a positive after-effect.

In this study, we tested the same group of participants on a TOJ and a SJ task and investigated the sequential effect paradigm in both tasks. Further, we manipulated the range of SOAs so that one set of stimuli was centered around *objective* simultaneity (SOA centered at 0 ms) or around the *subjective* simultaneity independently measured on a per individual basis (SOA centered at the individual’s PSS or iPSS). We first aimed at replicating the rapid recalibration effects previously reported with SJ and TOJ, notably the observation predicting that sequential effects would be opposite in the two tasks [30]. Second, we asked whether centering the range of SOAs on the objective or subjective clock would affect rapid recalibration effects. Finally, we evaluated whether, considering the same pool of participants and range of SOAs, the iPSS calculated with an SJ task would be identical to the one calculated with the TOJ task.

## Materials and methods

### Participants

Twenty participants (11 males, mean age: 23 ±3 y.o.) took part in the study. The sample size was determined based on previous studies [30]. All had a normal or corrected-to-normal vision, normal color vision, and normal hearing. All were naive as to the purpose of the study. Each participant provided written informed consent in accordance with the Declaration of Helsinki (World Medical Association, 2013), and the study was approved by the Human Research Ethics Committee at NeuroSpin (Gif-sur-Yvette, France).

### Stimuli

**Fig 1:**
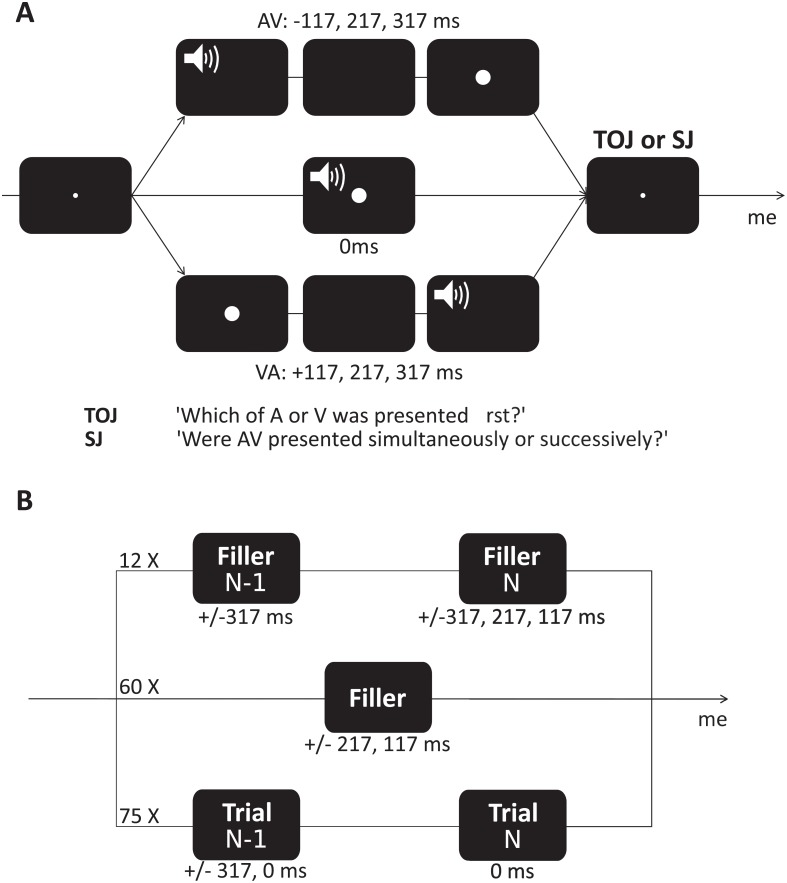
Experimental design. A: In both TOJ and SJ tasks, a trial started with a fixation point followed by the presentation of an auditory (A) and a visual (V) stimuli separated by variable delays (SOAs). SOAs were drawn from ±317, ±217, ±117, and 0 ms centered on the cSOA (cSOA could be objective (0 ms) or subjective simultaneity (iPSS)). SJ and TOJ were blocked. The main experimental question (bottom line) focused on the sequential effect of the AV delay in trial N-1 (simultaneous or large asynchrony) on participants’ temporal judgment in trial N. Filler trials tested other combinations in which the trial N-1 was SOA = ±317*ms* (first line) and trials equal to ±217*ms* and ±317*ms*. The inter-trial interval (ITI) was always 1.2s.

The experiment was designed using PsychToolbox (Version 3.0.11; http://psychtoolbox.org/ [31]) and written in Matlab (Version 2014a; The MathWorks, Inc., Natick, MA). Visual stimuli consisted of a white disk (diameter = 1.8°) presented for 16.7 ms (1 frame with a refresh rate of 60 Hz) on a black CRT screen placed 65 cm away from participants. The center of the visual stimuli was presented at an eccentricity of 14° of visual angle from the horizontal meridian. Auditory stimuli consisted of white noise presented for 16 ms (incl. 5 ms fade-in and fade-out) presented at a comfortable hearing level (65 dB) using earphones. Seven delays were tested separating A and V by ±317, ±217, ±117, or 0ms. A negative delay corresponded to the auditory target being presented first (Fig 1A).

### Procedure

Each experimental session consisted of six experimental blocks. In all blocks, participants were asked to perform two possible tasks: a temporal order judgment (TOJ) or a simultaneity judgment (SJ) task. In both tasks, participants were presented with a series of simultaneous or asynchronous audiovisual (AV) events. In TOJ, participants judged whether the sound or the flash appeared first. In SJ, participants judged whether the visual and the auditory events were simultaneous or not. Both tasks were a two-alternative forced-choice (2-AFC).

In the first two experimental blocks, participants completed a SJ and a TOJ with 70 trials each. AV delays were sampled equally from ±317, ±217, ±117, and 0ms (Fig 1A). These blocks were used to calculate the point of subjective simultaneity (iPSS) of each participant. We used the toolbox psignifit [32] to fit the psychometric curves with a cumulative normal. To calculate each individual’s PSS from the TOJ data (thereafter, iPSS), we used a threshold of 50%. To calculate the iPSS from the SJ data, we fitted a sigmoid curve on each side of the response distribution and used the average of their 75% estimated threshold. To estimate the goodness of individuals’ fit, we used a bootstrap approach as proposed in [33] in which the deviance of the fitted model is compared to deviances that were to be expected if the fitted model was perfectly correct. We used the proportion of the deviance values from simulations (from 5000 samples) that were greater than the observed deviance of the model (the greater this proportion, the better the fit). Given that for SJ we fitted two curves, we report the goodness of fit for both.

Following the assessment of each individual’s PSS, the experiment continued with four experimental blocks (2 SJ: one with cSOA = 0 ms, one with cSOA = iPSS; 2 TOJ: one with cSOA = 0 ms, one with cSOA = iPSS), each testing AV delays centered at 0ms (cSOA = 0 ms) or shifted at iPSS (cSOA = iPSS). One experimental manipulation indeed consisted of incorporating our knowledge of the individual PSS (iPSS) thereby yielded two kinds of SOAs distributions: in the *objective clock* blocks, we used SOAs selected from ±317, ±217, ±117, and 0ms with negative delays corresponding to the auditory event being presented first (Fig 1A). In the “subjective clock” blocks, we use the same SOAs this time centered around the iPSS of each participant so that the simultaneity condition (SOA = 0 ms in the *objective block*) was now the iPSS. The selected SOAs, in the *subjective clock* blocks, were thus selected from ±(317 + *iPSS*), ±(217 + *iPSS*), and ±(117 + *iPSS*). Each experimental block contained 234 trials, with an inter-trial interval (ITI) of 1.2s. For all blocks, we configured 75 pairs of trials so that the trial N with an AV delay equal to cSOA (0 ms or iPSS conditions) was preceded by the trial N-1 in which the AV delay was equally sampled from 0 ms or ±317*ms* (Fig 1B, top line). We included 60 filler trials in the design with delays of ±217 or ±117*ms* equally distributed in each experimental block (Fig 1B, middle row). Fillers were used to avoid habituation of participants and thus prevent biasing participants’ responses. Finally, we included 12 extra pairs of trials composed of a N-1 trial with a ±317*ms* AV delay followed by each of the possible AV delays from the set ±317,±217, and ±117*ms* (Fig 1B, bottom line). Half of the participants started with the TOJ blocks, and the other half with the SJ blocks.

### Statistical analysis

We performed repeated-measures ANOVAs, estimated effect sizes (omega-squared for ANOVAs) and performed post-hoc tests (using the Holm–Bonferroni method to correct for multiple comparisons) using JASP [34]. The p-values were adjusted, whenever appropriate, for violations of the sphericity assumption using Greenhouse-Geisser correction. We also included Bayesian ANOVAs and report the BF(inclusion), which compares two classes of models, one class with the factor of interest and one without.

## Results

### Inter-individual variability: iPSS in TOJ differ from iPSS in SJ

**Fig 2:**
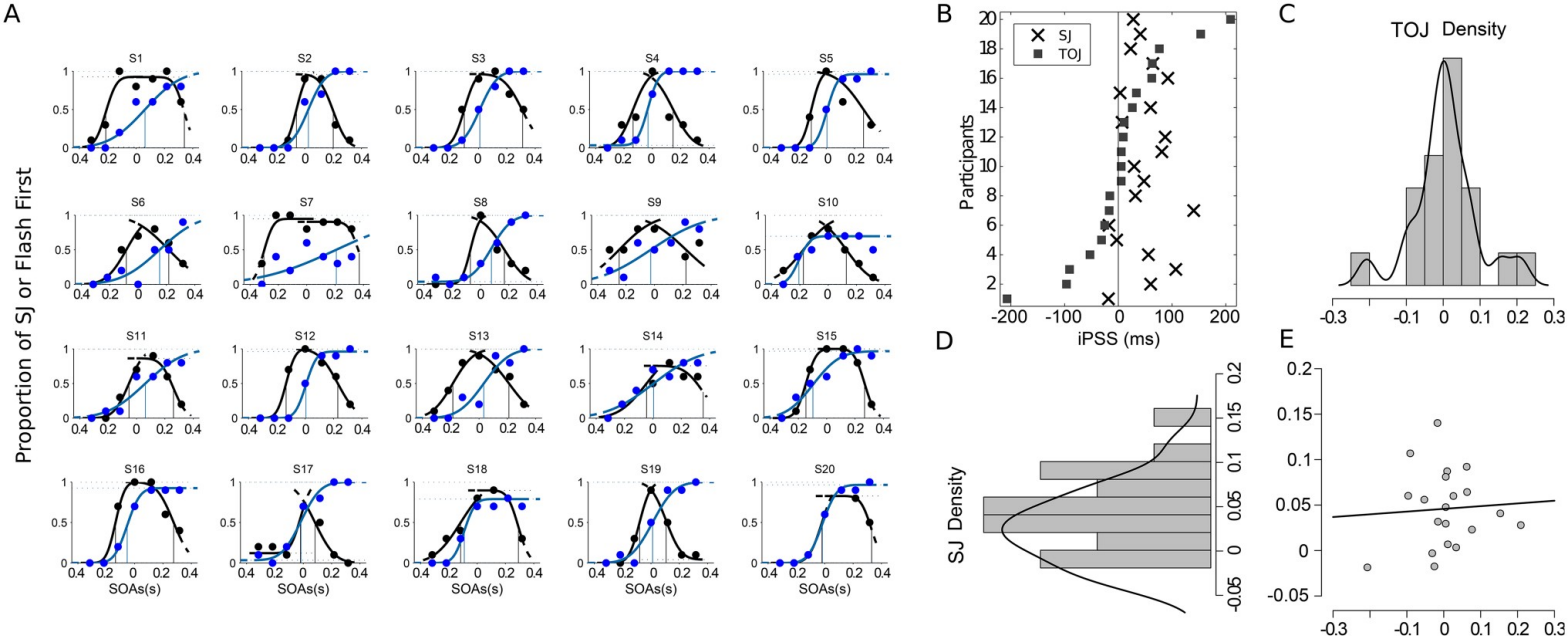
Comparison between the PSS calculated from the TOJ and the SJ tasks on a per individual basis. A: Individual psychometric curves for TOJ (blue) and SJ (black). B: Distribution of iPSS calculated from TOJ (square) and SJ (cross). C: Distribution of iPSS observed in the TOJ task. D: Distribution of iPSS in the SJ task. E: The correlation between iPSS in both tasks was not significant (Pearson r = 0.062, p = 0.794; Spearman correlation, rho = 0.025, p = 0.917).

First, we looked at the distribution of the iPSS in the TOJ and SJ tasks. Goodness of fit for both tasks were in general high (TOJ: mean = 0.74, range:[0.13 0.99], SJ left curve: mean = 0.73, range:[0.16 0.99], SJ right curve: mean = 0.72, range:[0.10 0.99]). We found a large inter-individual variability in the iPSS observed in the TOJ task (Fig 2A and 2B) with iPSS spanning from -200 to +200 ms. The distribution of iPSS in the SJ task showed a narrower spread with a bias towards positive AV delays, i.e., towards the sound being presented first to perceive AV simultaneity. The range of inter-individual variability in the TOJ task was consistent with a prior assessment of iPSS distribution in AV temporal order [35] and the lack of correspondence between the PSS in TOJ and SJ was also consistent with previous studies [20, 36–38].

As these observations seemed to indicate that the iPSS in TOJ and SJ tasks were different within and across individuals, we directly compared the two sets of iPSS values and found no significant correlations between the iPSS in TOJ and those in SJ (Pearson r = 0.062, p = 0.794, *BF*_10_ = 0.29; Spearman rho = 0.025, p = 0.917) (Fig 2E). A robust skipped-correlation (which protects against bivariate outliers) [39] also showed no significant correlation (r = -0.15, p = 0.088). Additionally, the PSS distributions were different for these two tasks (Fig 2C and 2D) with the SJ distribution skewed towards positive delays, contrarily to the TOJ distribution.

Considering that physical simultaneity may not be a reliable control from the perspective of the observer due to the iPSS being seldom observed at 0 ms of AV delay on a per individual basis, we explored and contrasted sequential effects in TOJ and SJ tasks using either a distribution of SOAs centered on physical simultaneity (cSOA = 0ms; objective clock) or on the subjective simultaneity (cSOA = iPSS; subjective clock).

### Positive sequential effects in TOJ

**Fig 3:**
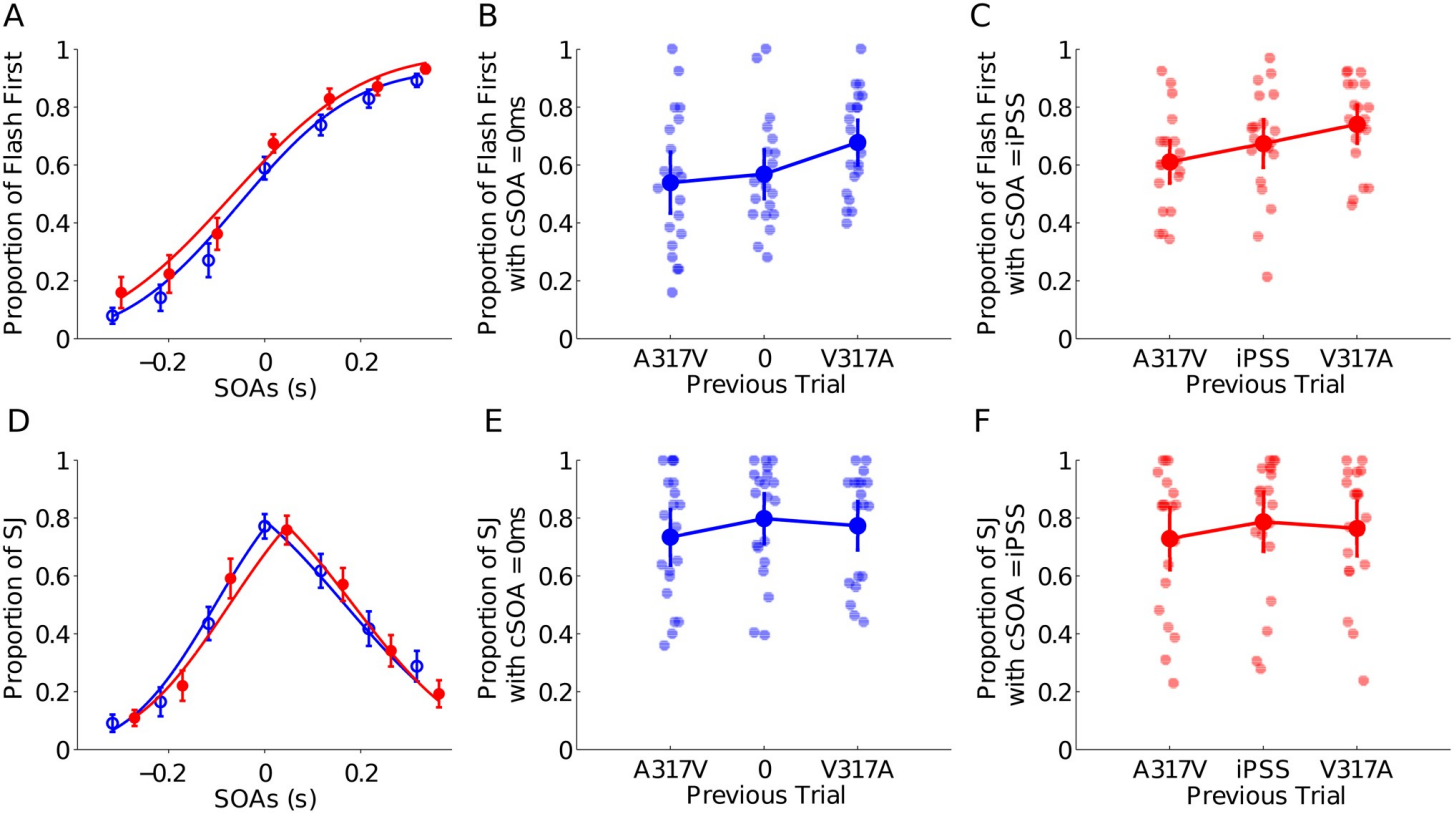
A: TOJ psychometric curves with cSOA equal to 0ms (blue) and iPSS (red). B: The dots with line show the mean proportion of “flash first” responses, with cSOA = 0ms, when the trial N-1 had SOAs equal to -317ms (A317V), 0ms, and 317ms (V317A). The background dots show the proportion of “flash first” responses of all participants in each condition. C: Same as B, with cSOA = iPSS. D: SJ psychometric curves with cSOA equal to 0ms and iPSS. The middle of the iPSS psychometric curves of TOJ and SJ (red curves) is the average of the participants’ iPSS in each task, and thus, both curves have been shifted. E: Same as B, for SJ task with cSOA = 0ms. F: Same as B, for SJ task with cSOA = iPSSms.

First, and irrespective of the cSOA (0 ms in blue, or iPSS in red), we observed similar psychometric curves (Fig 3A). The mean PSS was 6.17ms ±19.70ms, range between −207.04ms to 208.90ms.

To measure rapid sequential effects, we focused our analysis on trials in which AV stimuli were simultaneous either from the perspective of the *objective clock* or the *subjective clock* (i.e. 0 ms or iPSS, respectively. Fig 3B and 3C)). Simultaneous trials were preceded by trials with an SOA of -317ms, 0ms, or 317ms. The proportion of visual first responses were transformed using an arcsin square root transformation and submitted to a 2 x 3 repeated measures ANOVA with clock condition (*objective* or *subjective* clock) and trial N-1 (-317ms, 0ms, 317ms) as factors. We found a significant effect of the N-1 trial AV delay on the temporal order judgment of trial N (F(2,38) = 8.655, p = 0.003, *ω*^2^ = 0.272, BF(inclusion) = 11.7). A post-hoc analysis showed that when participants were exposed to a positive AV delay (visual first) on the trial N-1, they had a significantly higher proportion of visual first responses in trial N than when participants were exposed to a simultaneous trial N-1 (t(19) = -3.973, *p* < 0.001, d = -0.89), or to a negative delay (auditory first) (t(19) = -4.289, *p* < 0.001, d = -0.96). The comparison between the exposure to an auditory first and to simultaneous trials was however not significant (t(19) = -1.684, p = 0.30, d = -0.38). The effect of clock condition was also not significant (F(1,19) = 2.949, p = 0.102, *ω*^2^ = 0.085, BF(inclusion) = 1.437) and we found no interactions of clock type with trial N-1 (F(2,38) = 2.168, p = 0.128, *ω*^2^ = 0.054, BF(inclusion) = 0.372).

### Sequential effect in SJ

The simultaneity profiles in both tested cSOA were similar in the SJ task, with a small right shift of the simultaneous responses in the subjective clock towards positive SOA (Fig 3D). Accordingly, the mean iPSS was 46ms ±9.40ms, ranging between −18.60ms and 140.30ms.

As previously, we focused our analyses on trials in which AV stimuli were presented simultaneously (objective clock) or at participants’ iPSS (subjective clock, Fig 3E and 3F)) and that were preceded by trials with SOAs of ± 317ms or 0ms. The proportion of simultaneity responses was transformed using an arcsin square root transformation before submitting the data to a 2x3 repeated measures ANOVA with clock condition (objective or subjective) and N-1 trial (-317ms, 0ms, 317ms) as factors. We found no significant effect of trial N-1 on simultaneity judgments (F(2,38) = 2.903, p = 0.085, *ω*^2^ = 0.085, BF(inclusion) = 0.25), of subjective or objective clock condition (F(1,19) = 2.949, p = 0.102, *ω*^2^ = 0.085, BF(inclusion) = 0.14) or interaction of these two factors (F(2,38) = 2.168, p = 0.128, *ω*^2^ = 0.054, BF(inclusion) = 0.02).

## Discussion

In the present study, we investigated sequential effects in TOJ and SJ tasks and compared whether centering the range of presented SOAs on the objective or on the subjective clock modulated these effects. We found a rapid recalibration effect in TOJ, but not in the SJ task. Moreover, we found that centering the SOAs on objective or subjective simultaneity did not significantly change the pattern of results.

Several studies have shown that repeated exposure to specific delays between two stimuli can lead to negative after-effects in both TOJ and SJ tasks [22–25]. A growing number of studies have shown that a single exposure to a delay can lead to similar after-effects in SJ [26–29] whereas the number of studies that have investigated a similar effect in TOJ is still incipient [30, 40]. In both studies of sequential effects in TOJ, the authors reported that, contrary to what has been found in SJ tasks and to repeated exposure in TOJ tasks, a single exposure to a delay yields a positive after-effect [30, 40].

Our results in the TOJ task are in general agreement with these studies: we found a positive after-effect in which the proportion of “flash first” responses increased when the previous trials were a flash first pair. In contrast to previous findings [26–29], we did not find effects of previous trials in our SJ task. However our experimental design might not have been ideal to find rapid recalibration in SJ. In our task, our main condition of interest was in trials in which the physical interval between events was either at 0ms (objective clock condition) or the participants PSS (subjective clock condition). Although this might have decreased our power to detect differences in SJ, we believe this design might be useful in future neuroimaging studies in which having a large number of trials in a specific physical interval can help in critical comparisons.

It is still not clear why TOJ leads to different after-effects than SJ. In his paper, Roseboom [30] proposed three different mechanisms that could potentially explain after-effects in general: the local repulsion of timing, the assimilation of timing, or the assimilation of decision criteria. Importantly, he argues that none of these views is consistent with the full pattern of results found in rapid and repeated exposure of SJ and TOJ tasks [30].

A recent study, however, has suggested that a choice-repetition bias [40] could explain the contradictory results found in TOJ task. In fact, a choice-repetition could explain our findings for TOJ: if in trials in which participants have maximum doubt they repeat their last response, this should lead to an effect similar to what we have observed. On the other hand, in our experiment, the SJ was relatively easy, and, for this reason, participants might not have presented a similar bias.

Irrespective of the underlying mechanisms of the observed recalibrations, it could be expected that these would play a stronger role when presentations were at the subjective simultaneity of each participant. This should be true especially in TOJ, given that this would be the most ambiguous SOA, at which participants would be in maximal doubt as to their response choice. However, the pattern of recalibration did not depend on whether the SOAs were centered on physical simultaneity or participants’s subjective point of simultaneity. One possibility for this lack of effect is that the estimated points of subjective simultaneity were estimated with a small number of trials. Critically, although this might be hindering the impact of using physical or subjective simultaneity, the effects of temporal recalibration remained.

Finally, we found that the PSS estimated from SJ and TOJ tasks were different and not significantly correlated. This finding is in accordance with several results that suggest that these measures likely result from different underlying mechanisms [20, 36–38, 41].
